# Comparison of Microscopy and PCR-RFLP for detection of *Anaplasma marginale* in carrier cattle

**Published:** 2010-06

**Authors:** V Noaman, P Shayan

**Affiliations:** 1 Veterinary Department of Isfahan Research Center for Agriculture and Natural Resources, Isfahan, Iran; 2 Center for Ticks and Tick-borne Diseases, Faculty of Veterinary Medicine, University of Tehran, Tehran, Iran

**Keywords:** *Anaplasma marginale*, Carrier cattle, Microscopic method, Giemsa staining, PCR-RFLP

## Abstract

**Background and Objectives:**

In Iran, anaplasmosis is normally diagnosed with traditional Giemsa staining method. This is not applicable for identification of the carrier animals. The aim of this study was to compare the detection of *Anaplasma marginale* in two different numbers of microscopic fields (50 and 100) using conventional Giemsa staining method compared with the PCR-RFLP technique.

**Materials and Methods:**

In this study, examinations were performed on 150 blood samples from cattle without clinical signs. Sensitivity and specificity of two microscopic fields (50 and 100 fields) were compared with *A. marginale* specific PCR-RFLP. The degree of agreement between PCR-RFLP and the two microscopic tests was determined by Kappa (κ) values with 95% confidence intervals.

**Results:**

PCR-RFLP showed that 58 samples were *A. marginale*, while routine microscopy showed erythrocytes harboring *Anaplasma* like structures in 16 and 75 blood samples determined in 50 and 100 microscopic fields respectively. Examination of 50 and 100 microscopic fields showed 25.8% and 91.4% sensitivity and 99% and 76.1% specificity compared to 100% sensitivity and specificity by PCR-RFLP. The Kappa coefficient between PCR-RFLP and Microscopy (50 fields) indicated a fair level of agreement (0.29). The Kappa coefficient between PCR-RFLP and Microscopy (100 fields) indicated a good level of agreement (0.64)

**Conclusion:**

Our results showed that the microscopic examination remains the convenient technique for day-to-day diagnosis of clinical cases in the laboratory but for the detection of carrier animal with low bacteremia, microscopy with 100 fields is preferable to Microscopy with 50 fields and molecular methods such as PCR-RFLP can be used as a safe method for identifying cattle persistently infected with *A. marginale.*

## INTRODUCTION

Anaplasmosis is an arthropod-born disease of cattle and other ruminants caused by species of the genus *Anaplasma* (Rickettsiales: Anaplasmataceae) (1). Four species, including *Anaplasma marginale*, *A. centrale*, *A. bovis* and *A. phagocytophilum* are recognized in blood of Iranian cattle by molecular methods (2–5). Based upon location within the infected erythrocyte, two species of *Anaplasma* that infect cattle have been described, *A. marginale* and *A. centrale*. In addition to having differences in morphology, these species display differences in virulence and geographical distribution. *A. centrale* causes mild infections in cattle. In contrast, the closely related rickettsia *A. marginale* is the aetiological agent of acute anaplasmosis, a bovine syndrome characterised by a progressive haemolytic anaemia associated with fever, weight loss, abortion, decreased milk production and in some cases, death of the infected cattle (6).

Anaplasmosis caused by *A. marginale* is an economically important and widespread disease of cattle in most tropical and subtropical countries, including Iran (7, 8). The infectious agent transmitted either biologically by ticks or mechanically by other arthropod vectors (9). Following transmission, *A. marginale* invades and multiplies within mature erythrocytes. During acute anaplasmosis, rickettsemia levels exceed 10^9^ infected erythrocytes per ml and the resulting disease is characterized by anemia, weight loss, abortion, and death (10). Recovery from acute anaplasmosis results in persistent infection characterized by repetitive cycles of rickettsemia ranging from approximately 10^2.5^ to 10^7^ infected erythrocytes per ml (10). Persistently infected cattle serve as long-term reservoirs for transmission within herds (7). Detection of persistently infected cattle is important to control the movement of infected cattle into and from disease-free regions.

Conventional method for identification includes examination of blood smears using Giemsa staining, which is accompanied with some critical problems. Diagnosis of *A. marginale* is performed routinely by morphological identification based on location of inclusion bodies marginally within the erythrocytes (11). Microscopic examination by Giemsa staining of blood smears can only detect levels of >10^6^ infected erythrocytes per ml (12). Giemsa-stained blood smears can be indeed used as a suitable method to detect *Anaplasma* agents in the animals clinically suspected acute anaplasmosis, but it is not applicable for the determination of pre-symptomatic or carrier animals (13).

Conventional microscopy is time-consuming and tedious. Furthermore, microscopic examination of Giemsa stained blood smears, especially from carrier animals, is accompanied with several problems. First of all, due to the very low amount of infected erythrocytes in carrier animals, the detection of good stained *Anaplasma* organism is very limited and the microscopy it is not possible to distinguish between *A. marginale* and *A. centrale*. Additionally the differentiation between *Anaplasma* organisms and structures like Heinz bodies, Howell-Jolly bodies or staining artifacts, which often seen in Giemsa stained blood smears need special experiences (14).

Molecular methods, with a high degree of sensitivity and specificity, have been developed to identify *A. marginale* DNA (13, 15) (16) and polymerase chain reaction (PCR) assay has been considered the “gold standard” for detection of persistently infected cattle (17).

In Iran, anaplasmosis is normally diagnosed with the traditional Giemsa staining method, yet it seems not to be applicable for identifying of the carrier animals (8). The aim of this study was to compare the detection of *Anaplasma* organisms in two different numbers of microscopic fields (50 and 100) using conventional Giemsa staining method with the PCRRestriction Fragment Length Polymorphism (RFLP) technique.

## MATERIALS AND METHODS

**Collection of blood Samples.** From June 2007 to October 2007, 30 farms in Isfahan province, central part of Iran, were selected for the study based on their history of outbreak of bovine anaplasmosis. Blood samples were collected from jugular vein of 150 Friesian and crossbred cattle ranging between 1 and 9 years. Five hundred micro liters of each collected blood samples was fixed with 1 ml 96% ethanol in 1.5 ml sterile eppendorf tubes. Additionally, two thin blood smears were prepared immediately after each blood collection. The blood smears were air dried, fixed in methanol, stained with Giemsa and analyzed for the presence of *A. marginale* in the erythrocytes at 100× magnification. In each blood smear both 50 and 100 fields were examined separately by a single observer. All smears carefully examined to estimate the Percent Parasitized Erythrocytes (PPE) as described by Coetzee *et al*. (2005) (18).

**DNA extraction.** DNA was extracted using a DNA isolation kit (MBST, Iran) according to the manufacturer's instructions. Briefly, ca. 5 mm^3^ big pieces of fixed blood samples were first air dried and subsequently lysed in 180 l lysis buffer and the proteins were degraded with 20 l proteinase K for 10 min at 55°C. After addition of 360 l Binding buffer and incubation for 10 min at 70°C, 270 l ethanol (96%) was added to the solution and after vortexing, the complete volume was transferred to the MBST-column. The column was first centrifuged, and then washed twice with 500 l washing-buffer. Finally, DNA was eluted from the carrier using 100 l Elution buffer. The amount of extracted DNA and its purity was measured by OD^260^ and the ratio of OD^260^ to OD^280^ respectively. In addition the extracted DNA was analyzed on agarose gel before use.

**PCR.** Primers were designed from the published sequence of 16S ribosomal RNA (GenBank accession no. M60313) from *A. marginale* and were as follows P1 (Forward, 5'AGAGTTTGATCCTGGCTCAG3‘, positions 1 to 20); P2 (Reverse, 5'GTTAAGCCCTGGTATTTCAC3’, positions 558 to 577). Approximately 100 to 500 ng DNA was used for the PCR analysis. The PCR was performed in 100 l total volume including one time PCR buffer, 2.5 U *Taq* DNA Polymerase (Cinnagen, Iran), 2 l of each primer (P1/P2, 20 M, Cinnagen, Iran), 200 M of each dATP, dTTP, dCTP and dGTP (Fermentas, EU) and 1.5 mM MgCl2 in automated Thermocycler (MWG, Germany) with the following program: 5 min incubation at 95°C to denature double strand DNA, 35-38 cycles of 45 s at 94°C (denaturing step), 45 s at 56°C (annealing step) and 45 s at 72°C (extension step). Finally, PCR was completed with the additional extension step for 10 min. The PCR products were analyzed on 2% agarose gel in 0.5 times Tris-Borate-EDTA (TBE) buffer and visualized using ethidium bromide and UV-illuminator. A molecular mass ladder (100 bp) and positive and negative controls were used for each batch run. Each sample was spiked with positive control *A. marginale* DNA to detect any inhibition of the PCR that might lead to falsenegative results.

**RFLP for *A. marginale.*** One micro liter of the extracted DNA from blood samples was amplified with the primers P1/P2, resulting in a PCR product of 577 bp for all *Anaplasma* spp. The PCR products were purified from enzyme and salts using PCR-product purification kit (MBST). 10 l of purified PCR product (577 bp) was then cut with 0.1 l restriction endonuclease *Bst* 1107 I (Roche, 10U/l) in 2.5 l 10 x corresponding buffer and 12.5 l H_2_O for 1 h by 37°C. As control 10 l PCR products was treated with 2.5 l 10 x corresponding buffer and 12.5 l H_2_O without adding of enzyme.

**Statistical analysis.** The degree of agreement between PCR-RFLP and the two microscopical tests was determined by Kappa (κ) values with 95% confidence intervals. We used the PCR-RFLP as the reference test to calculate the relative sensitivity and relative specificity of the microscopical tests.

## RESULTS

**Fig. 1:**
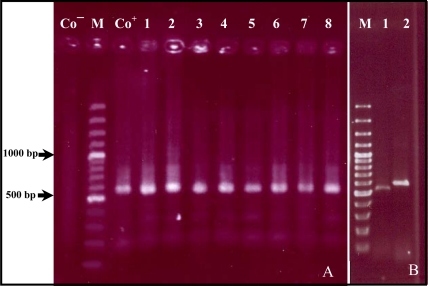
DNA isolated from blood was analysed by PCR and PCR-RFLP. **A:** DNA was amplified with primer P1/P2 resulting in PCR product of 577 bp in length (lanes 1-8). **B:** PCR product of 577 bp (line 2) was cut with restriction endonuclease. BST1107I resulting in DNA fragment of 509 bp (line 1). Co^–^ =Negative control. Co+ =Positive control. M=100 bp molecular marker.

The DNA was extracted from blood samples and analyzed by PCR using primers derived from the 16S rRNA gene. The nucleotide sequence of 16S rRNA gene is highly conserved in *Anaplasma* spp*.* and the primers P1/P2 can amplify the corresponding fragments of the gene in all known *Anaplasma* species. PCR analysis of the DNA isolated from blood samples showed that 58 out of the total 150 blood samples were *Anaplasma* spp*.* positive and revealed an expected PCR product of 577 bp in length (Fig. 1A).

For determination of *A. marginale* specificity of the PCR products, PCR-RFLP method was used (2, 3). The restriction endonuclease *Bst* 1107I recognizes the sequence (GTATAC) in corresponding PCR product (577 bp) of *A. marginale* and makes a cut in position 68, whereas the used restriction enzyme can not cut the corresponding PCR product of other *Anaplasma sp*. Analysis of all 58 *Anaplasma* positive PCR products with the restriction endonuclease *Bst* 1107I showed that all PCR products could be cut in two expected DNA fragments with 509 bp and 68 bp in length respectively (Fig. 1B). Fifty-eight blood samples were *A. marginale* positive by PCR-RFLP. Ninety-two samples were *Anaplasma* negative by PCR-RFLP.

Interestingly, Giemsa staining analysis of blood smears showed different results dependent upon the chosen number of examined microscopic fields by 100 x magnification. In 16 out of 150 blood samples, *Anaplasma* like structures could be identified when the examination was performed in 50 microscopic fields. From these 16 samples, 15 were also PCR-RFLP positive and 1 sample was negative. When the examination was performed in 100 microscopic fields, 75 samples were determined as *Anaplasma* positive, from which 53 samples were also PCR-RFLP positive. This means that 22 samples were *Anaplasma* false positive (Table 1).

**Table 1:** Comparison of results of PCR-RFLP assay and microscopic examination for *A. marginale* in 150 cattle blood samples.

PCR-FLP assay results	Microscopy results				

	50 fields			100 fields	
		
	+	−		+	−
+	15	43		53	5
−	1	91		22	70

The percentage of erythrocytes harboring *Anaplasma* like structures varied in the positive blood samples from 10-3% to 10-2%. Examination of 50 and 100 microscopic fields showed 25.8% and 91.4% sensitivity and 99% and 76.1% specificity respectively compared to 100% sensitivity and specificity for PCR-RFLP (Table 2).

**Table 2:** Sensitivity and specificity of microscopical methods compared to 100% sensitivity and specificity of PCR-RFLP for detection of *A. marginale* in carrier cattle.

Method	No. of samples examined	No. of positives detected	Sensitivity^a^(%)	Specificity^b^(%)
PCR-RFLP	150	58	100	100
Microscopy(50 fields)	150	16	25.8	99
Microscopy(100 fields)	150	75	91.4	76.1

a Calculated as follows: [number of true positives/(number of true positives + number of false negatives)] × 100.

b Calculated as follows: [number of true negatives/(number of true negatives + number of false positives)]× 100.

The Kappa coefficient between PCR-RFLP and Microscopy (50 fields) indicated a fair level of agreement (0.29). The Kappa coefficient between PCR-RFLP and Microscopy (100 fields) indicated a good level of agreement (0.64). The Kappa coefficient between Microscopy with 100 fields and Microscopy with 50 fields indicated a poor level of agreement (0.2).

## DISCUSSION

Molecular methods based on DNA with high degree of sensitivity and specificity have been developed (13, 16). PCR methods based on the 16S rRNA gene are already used for differentiation of genus *Anaplasma* (11) but the sequence strong similarity of this gene in *A. marginale* and *A. centrale* do not allow the use of the simple PCR method for discrimination between these two species. The 16S rRNA sequence of *A. marginale* and *A. centrale* differed only in two positions within hyper-variable region (V1) and designing of species-specific primers is near impossible (2).

Microscopic examination of Giemsa stained blood smears were used traditionally to diagnose not only the acute anaplasmosis but also to detect the carrier animals in Iran, which is accompanied with serious problems. Serological tests were also developed for the diagnosis of anaplasmosis. But due to the cross reactivity, this method is not suitable for the differential diagnosis of anaplasmosis (19–23).

Our results showed that the traditional microscopic examination of blood smears is not able to detect low bacteremia in carrier cattle. Furthermore, the *Anaplasma* like structures recognized in erythrocytes are often difficult to differentiate from Heinz bodies, Howell-Jolly bodies or staining artifacts (14). This means, due to the very low amount (10^-2^% – 10^-3^%) of infected erythrocyte in the examined carrier cattle, it is very difficult to determine the *Anaplasma* organisms by simple Giemsa staining, which is performed routinely in the laboratories in Iran. To determine the sensitivity and specificity of the microscopic examination, 50 and 100 microscopical fields were analyzed and compared with the corresponding PCR-RFLP analysis.

Examination of 50 microscopic fields showed 25.8% sensitivity and 99% specificity compared to 100% sensitivity and specificity of PCR-RFLP. With sole use of this method, 16 blood samples were identified as *Anaplasma* positive, from which 1 sample was false positive. This means that within 150 blood samples, 43 PCR-RFLP positive samples were recognized as false-negative. In Iran, most of the veterinary laboratories examine 50 microscopic fields for detection of parasites in blood smears. Although this microscopic screening is specific, this approach often lacks the desired sensitivity.

Examination of 100 microscopic fields showed 91.4% sensitivity and 76.1% specificity compared to RFLP-PCR results. With this approach, 75 blood samples were recognized as *Anaplasma* positive from which only 53 samples were *Anaplasma* PCR-RFLP positive. This means that with this method, 22 blood samples were recognized as false positive samples. Although sensitivity of blood sample examination in 100 microscopic fields is greater than in 50 microscopic fields, but examination 100 microscopic fields yields lower specificity. This means that due to the very low amount (10^-2^%-10^-3^%) of infected erythrocytes in carrier cattle, it is very difficult to determine the *Anaplasma* organisms in the carrier cattle by simple Giemsa staining.

The agreement between PCR-RFLP and Microscopy with 50 fields was fair and the agreement between PCR-RFLP and Microscopy with 100 fields was good. Detection of anaplasma microscopically requires high bacteremia, good smear preparation, proper staining and a well-trained microscopist (in spite of the fact that the technique is cheaper and easier to perform). However, microscopic examination remains the convenient technique for day-to-day diagnosis of clinical cases in the laboratory.

We believe that the microscopic examination can fulfill the desired results for the diagnosis of acute anaplasmosis but for the detection of carrier animal with low bacteremia, Microscopy with 100 fields is preferable to Microscopy with 50 fields. Our results showed that for the detection of cattle infected persistently with *A. marginale,* the PCR-RFLP can be used as a safe method.
